# Rapid Development of Primary Right Atrial Angiosarcoma

**DOI:** 10.7759/cureus.64273

**Published:** 2024-07-10

**Authors:** Sophie Talbot, Vishal Bandaru, Tung Nguyen, Dauod Arif, Pooja Sethi

**Affiliations:** 1 Cardiology, Texas Tech University Health Sciences Center, Lubbock, USA; 2 Pathology, Texas Tech University Health Sciences Center, Lubbock, USA

**Keywords:** transesophageal echocardiogram, cardiac tamponade, pericardial effusion, cardiac angiosarcoma, primary cardiac tumor, exudative fluid analysis

## Abstract

Cardiac angiosarcomas are rare and generally followed by a high level of metastasis with poor median survival outcomes. Echocardiograms, CT scans, and MRIs are the standard methods for finding sites of cardiac tumors; however, immunohistochemical confirmation is necessary for a definitive diagnosis of angiosarcoma. A 58-year-old male presented to the emergency room with one week of dyspnea on moderate exertion accompanied by chest pain and alleviated with rest. A workup done to evaluate mass found a single 5 x 3.5 x 4.8 cm mass heavily vascularized by the right coronary artery and left circumflex involving the free wall of the right atrium with no extension to the tricuspid valve. Surgical resection was performed, and immunohistochemistry was consistent with a primary cardiac angiosarcoma. An exudative fluid analysis on pericardial and pleural fluid analysis may warrant screening for malignancy more frequently in concurrence with a patient’s history and presentation. Although the time from onset of symptoms to diagnosis of cardiac angiosarcoma is not well established, further investigation of such correlation may offer insight into survival post-treatment.

## Introduction

Cardiac angiosarcomas are a small segment of all soft tissue sarcomas, consisting of between 1% and 2% of the sarcomas. Unfortunately, the presentation of an angiosarcoma is also generally followed by a high level of metastasis that can range in incidence from 16% to 44% with poor median survival outcomes between six and 16 months for angiosarcomas in general and between 22 and 25 months for cardiac sarcomas [1-3]. One-, three-, and five-year survival rates of cardiac angiosarcomas have been reported to be 39%, 9%, and 8%, respectively, while five-year survival rates for angiosarcomas overall have been reported at 35% [4,5]. Of malignant neoplasms of the heart, angiosarcomas are the most common and comprise 10%-15% of primary cardiac malignancies [6]. The vast majority, approximately 80%, of cardiac angiosarcomas are found by the atrioventricular groove in the right atrium, commonly invading the lateral free wall myocardium and epicardium extending to the pericardium and sparing the septum [7-9]. Of the 25% of cardiac tumors that are malignant, sarcomas comprise approximately 95% of those cases and primary cardiac angiosarcomas make up approximately 30% within the 95% of cases [10]. There is a gender bias in the frequency of cardiac angiosarcomas where between two to three males are affected for every single female that is affected [11].

While symptoms of angiosarcomas may be dependent on the size of the tumor and the time for growth, overarching symptoms include chest pain, dyspnea, fatigue, weight loss, and malaise [11]. More severe symptoms may also develop as arrhythmia and surgical intervention may be necessary if pericardial effusion or cardiac tamponade occurs. Generally, echocardiograms have particularly high sensitivity for finding primary cardiac angiosarcomas, but CTs and MRIs remain the standard method to find the sites of tumors.

Transthoracic echocardiogram (TTE) specificity has been reported to be between 75% and 93.3%, while transesophageal echocardiogram (TEE) specificity has been reported at 96.8% [12,13]. X-rays may also be used, but regardless of the modality of diagnosis, immunohistochemical confirmation is necessary to confirm the diagnosis. Markers of a primary cardiac angiosarcoma include pathology positive for CD31, CD34, ERG, and factor VIII [11]. CD31 is a transmembrane glycoprotein adhesion molecule, expressed by platelets, megakaryocytes, and endothelial cells [14]. CD34 is a cell surface marker expressed in endothelial cells and hematopoietic stem cells prevalent in areas of enhanced vascular differentiation, although less sensitive than CD31 for angiosarcoma [11,14]. ERG is a member of the erythrocyte transformation-specific (ETS) family of transcription factors and is expressed in endothelial cells [14]. The direct causes for angiosarcomas are not typically known, but current research believes that there is a possible influence or causation from radiation, chronic lymphedema, genetic factors, and environmental conditions.

Treatment options almost always recommend cardiac resection. Without surgical resection, mean survival rates are approximately four to six months [11]. There is conflict on what chemotherapeutic and radiation treatments have the best effects, but generally, there is consensus that multimodal treatment has the best results [2,15]. Post-operative complications of cardiac sarcoma resections have included low cardiac output associated with higher rates of mortality, acute kidney injury, bleeding, sepsis, tachycardia, pneumothorax, delirium, and non-inclusive mesenteric ischemia [2].

## Case presentation

A 58-year-old male with a past medical history of hepatitis C, atrial fibrillation on apixaban, grade 1 diastolic congestive heart failure (CHF) with a left ventricular ejection fraction (LVEF) of over 70%, hypothyroidism, and large pericardial effusion with tamponade status-post subxiphoid pericardial window presented to the emergency room in November 2022 with one week of acute onset of shortness of breath on moderate exertion accompanied by chest pain and alleviated with rest. The patient was previously admitted in May 2022 for pericardial effusion with tamponade and underwent a pericardial window. A 40 ml pericardial fluid specimen was obtained, and cytology was negative for malignancy. A pericardial biopsy showed fibroconnective tissue with acute and chronic inflammation. This finding was presumed to be due to pericarditis, and the patient was treated with colchicine. Post-pericardial window, the patient developed atrial fibrillation and was started on apixaban with improvement. Transthoracic echocardiogram (TTE) done in May 2022 did not show signs of mass.

In November 2022, initial computed tomography (CT) angiography chest showed findings concerning for a right atrial mass measuring approximately 4 cm, as well as a moderate right pleural effusion. The initial lab workup was unremarkable, troponin was negative and flat at 13, and pro-brain natriuretic peptide (pro-BNP) was within normal limits. The patient was transferred for a higher level of care and cardiovascular surgery evaluation. A right thoracentesis was performed, and pleural fluid analysis noted pleural fluid protein 4.3 to serum 6.2, with pleural fluid lactate dehydrogenase (LDH) at 1,240, indicative of exudative pleural fluid (Table 1).

**Table 1:** Pleural Fluid Analysis RBC: red blood cell, WBC: white blood cell, LDH: lactate dehydrogenase.

	Pleural Fluid	Serum	Pleural Fluid-to-Serum Ratio
Color	Red	-	-
Clarity	Turbid	-	-
RBC count	2,212,000	-	-
WBC count	3,100	-	-
Neutrophils	6	-	-
Lymphocytes	14	-	-
Monocytes	6	-	-
Eosinophils	0	-	-
Basophils	0	-	-
Bands	0	-	-
Macrophages	31	-	-
Mesothelial	43	-	-
Fluid culture	No growth	-	-
Protein	4.3	6.2	0.69
LDH	1,240	432	2.87
Glucose	64	88	-
Amylase	11	-	-
Albumin	2.6	3.7	-

**Figure 1:**
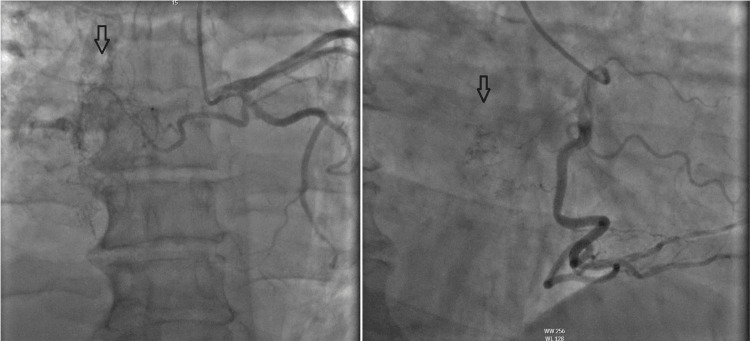
Left heart catheterization depicting a right atrial mass (arrow) with vascularization through small branches coming from both the left circumflex artery and the right coronary artery, respectively.

Left heart catheterization (LHC) showed prevalent atrial branches from both the right coronary artery (RCA) and left circumflex artery (LCX) system that appeared to fill a heavily vascularized structure in the vicinity of the right atrium. A CT chest/abdomen/pelvis was done and showed a right atrial mass incompletely evaluated on the current exam, moderate right pleural effusion along with hypo-ventilatory changes in the right lower lobe, scattered airspace opacities in the lungs compatible with infection, and fatty liver (Figure 1).

**Figure 2:**
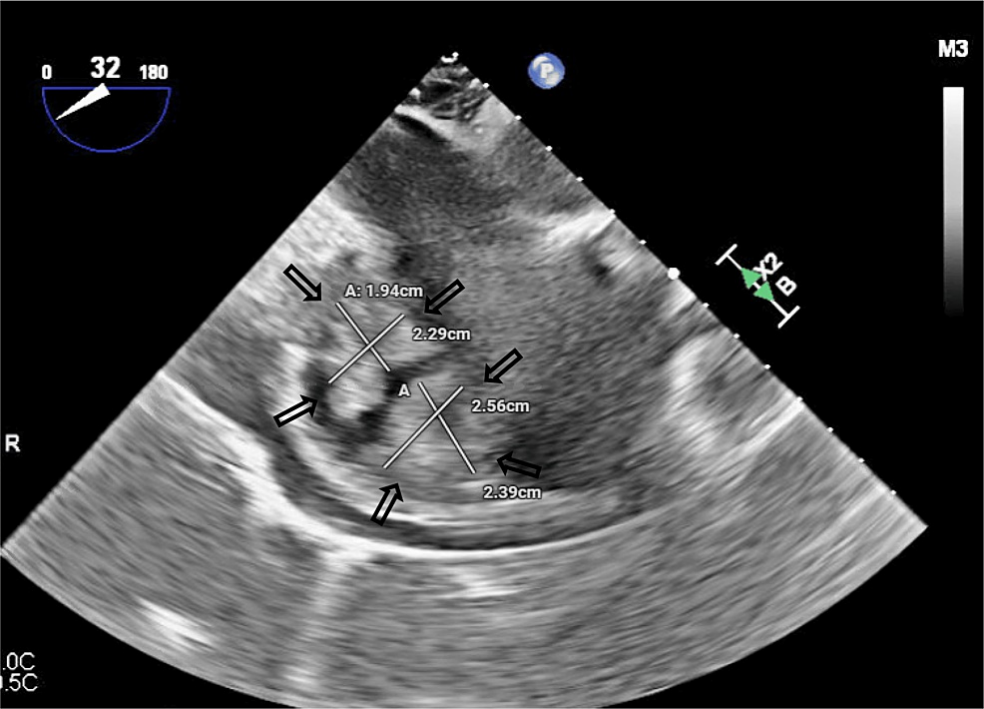
TEE with right atrial mass (arrows) with hyperechoic core and hypoechoic filamentous fronds attached to the mass suggestive of significant vascularization. Mass is closer to the SVC and thought to be either one large entity measuring up to 4.2 x 2.4 cm or two distinct adjacent 2 x 2 cm masses. TEE: transesophageal echocardiogram, SVC: superior vena cava.

A TEE with Doppler/color/contrast was then performed to evaluate possible mass versus thrombus which showed an atrial mass noted in the right atrium closer to the superior vena cava (SVC) thought to be either one large entity measuring up to 4.2 x 2.4 cm or two distinct adjacent 2 x 2 cm masses. The mass appeared to have a hyperechoic core with hypoechoic filamentous fronds attached to the mass and took up the echocardiogram contrast suggestive of significant vascularization. The findings were concerning for non-cardiac malignancy versus other highly vascularized masses (Figure 2).

**Figure 3:**
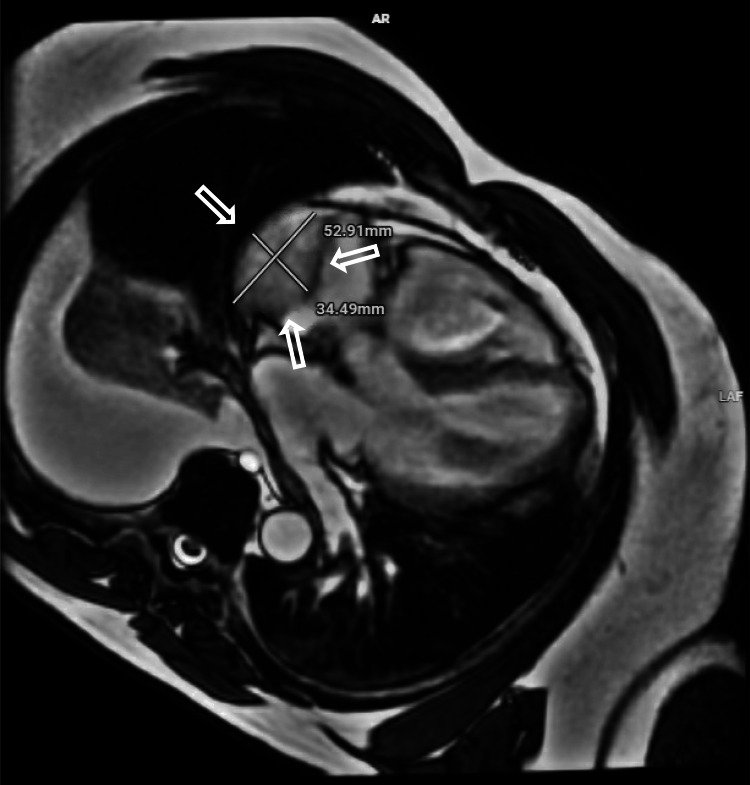
MRI of the chest with a single 5 x 3.5 x 4.8 cm mass (arrows) involving the free wall of the right atrium. No involvement of the tricuspid valve was found. MRI: magnetic resonance imaging.

Magnetic resonance imaging (MRI) was done to further evaluate mass, and findings showed a single 5 x 3.5 x 4.8 cm mass involving the free wall of the right atrium with no extension to the tricuspid valve. The imaging findings were most suggestive of a neoplastic process like a sarcoma, not compatible with a thrombus (Figure 3). 

**Figure 4:**
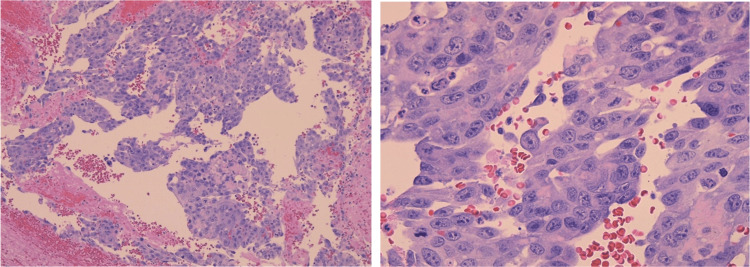
H&E stain at 10x and 40x, respectively, depicting irregularly shaped anastomosing vascular channels lined by atypical endothelial cells which are polygonal-epithelioid in shape and mitotically active. H&E: hematoxylin and eosin.

**Figure 5:**
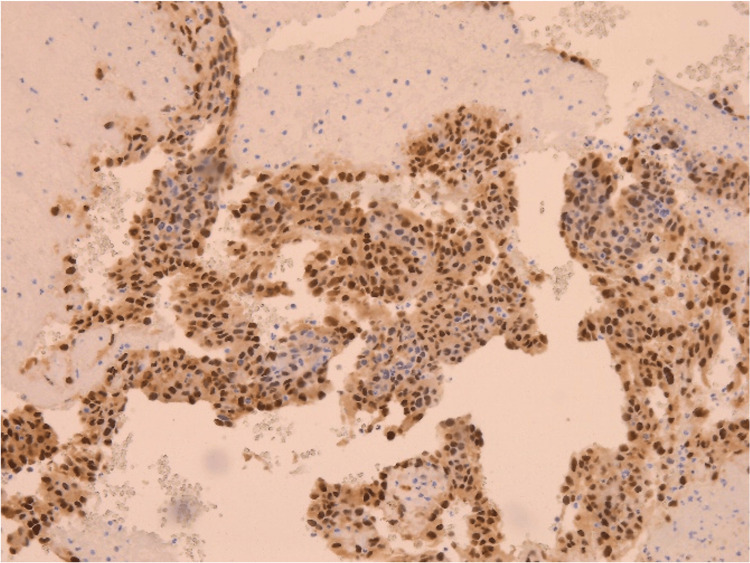
Immunohistochemical stain of biopsied mass is ERG positive, a marker for vascular tumors. ERG: ETS-related gene, a vascular marker (ETS: erythrocyte transformation-specific transcription factor).

The patient underwent sternotomy with mass resection. Much of the right atrial free wall was resected and reconstructed with a pericardial patch. The mass resection was sent to pathology and positive for malignant neoplasm with vascular differentiation, most consistent with angiosarcoma. The mass section showed spindle-shaped, polygonal, and epithelioid tumor cells, which were mitotically active (high Ki67 index) in solid and anastomosing vascular channels in some areas (Figure 4). The tumor cells were positive for CD31, CD34, and WT1. Immunohistochemistry (IHC) was ERG positive (a vascular marker) and negative for SOX10, pancytokeratin, synaptophysin, calretinin, CK 5/6, p40, TTF1, Ber-EP4, HHV8, Desmin, and D2-40 (Figure 5). Following stabilization of the patient status-post sternotomy with resection, the patient was discharged home in early December 2022.

The patient was readmitted shortly after discharge with a recurrent pleural effusion post-op and was treated with chest tube placement. Medport was placed for chemotherapy, and the patient was referred to oncology for further treatment and staging.

## Discussion

Clinical presentation of cardiac sarcomas is unpredictable, although common presentations include arrhythmias, diastolic congestive heart failure, and non-specific general symptoms such as dyspnea [16]. Similarly, cardiac tamponade has occurred concurrently with cardiac angiosarcomas [17]. However, on the initial echocardiogram, there was no indication of angiosarcoma as it was likely too small to be traced or the image resolution was too low to detect a mass. The development of atrial fibrillation is common after cardiac surgery; however, it is possible that the patient’s atrial fibrillation developed secondary to the angiosarcoma or was simply undiagnosed paroxysmal atrial fibrillation [18]. The patient developed these symptoms after he underwent a subxiphoid pericardial window so cardiac surgery is the most likely explanation.

Pleural fluid analysis can be a key indicator of etiology and possible diagnoses. An exudative pleural effusion is suggestive of malignancy [19]. Light’s criteria classify exudative and transudative pleural effusions based on the criteria of either a high pleural to serum LDH at a ratio above 0.6 or high pleural to serum total protein at a ratio above 0.5. The LDH ratio in November was 0.69 which fulfills light’s criteria for exudative pleural effusion. However, cytology was negative for malignancy. Pericardial fluid cytology in May was also negative for malignancy. The angiosarcoma may have still been present at the time of the initial ED visit in May but was not found due to the negative TTE and negative cytology, leading to the initial diagnosis of pericarditis. Pericardial and pleural effusion specificity and sensitivity vary a lot based on the type of malignancy and the type of criteria that are being evaluated. As such, classifying the presence of exudates is difficult in the context of malignancy due to the small number of patients in the category of cardiac angiosarcoma [19]. Utilizing case studies and retrospective studies also becomes difficult due to biases in presentation and whether they holistically represent the set of primary cardiac malignancies. More recent publications have analyzed cardiac angiosarcomas noting that a diagnosis of angiosarcoma based on exudative fluid analysis is rare and the specificity is incredibly variable between 40% and 90% based on a myriad of factors [20]. Many patients with cardiac angiosarcomas will receive their diagnosis post-mortem which begs the question of bias for successful pericardial fluid analysis.

Interestingly, very few studies investigate the past medical history of patients affected by this cancer in significant depth, possibly due to the lack of data [13,14]. The pathogenesis is not very clear in most cases, but there have been associations with radiation, chronic lymphedema, environmental carcinogens, and genetic syndromes such as xeroderma pigmentosa or bilateral retinoblastoma [1].

The current modalities of treatment are limited, and multimodal treatments have the best outcomes: surgery, radiotherapy, chemotherapy, targeted therapy, and immunotherapy. The baseline treatment for primary angiosarcomas is generally radical surgery [21]. Radiotherapy has been a good surrogate for older patient populations when surgery is not as viable of an option. Chemotherapy agents are generally required as the high metastatic rate of angiosarcomas will not subside post-surgical interventions. Angiosarcoma can develop incredibly quickly. From May to November, the right atrial angiosarcoma became diagnosable via TEE.

While targeted therapy and immunotherapy are promising alternatives, they are not nearly as widely used; however, both VEGF-targeted therapy (originally known as vascular permeability factor (VPF)), adrenergic receptors, and programmed death ligands and receptors are being evaluated and studied to create better clinical outcomes [1]. As advancements in medicine continue to decrease mortality rates, more lethal cancers such as atrial angiosarcomas may have new treatment options such as targeted therapy and immunotherapeutics as mentioned before.

## Conclusions

Primary cardiac angiosarcoma is a rare disease, often occurring in the right atrium, with an incredibly high mortality rate due to invasiveness, late diagnosis, rapid onset, and metastasis. In the case that we present, the angiosarcoma was first found and diagnosed on TTE six months after the patient’s pericardial tamponade, showing this rapid progression. Even with TTE and pericardial fluid analysis, early stages of angiosarcomas can be missed due to low resolution, the initial small size of the angiosarcoma, and low specificity and differing criteria for malignancy on cytology. A clear etiology of pericardial effusion, especially with signs of exudative fluid, may warrant screening for cancer more rigorously. Although the specificity of TTEs is often considered high for angiosarcomas, multimodal imaging such as an additional CT chest or MRI may have assisted in finding the angiosarcoma sooner. With many diagnostic and treatment challenges, radical resection of the primary tumor remains the most important and effective approach for optimal survival of patients diagnosed with early-stage cardiac angiosarcoma without evidence of metastasis.
